# Flow Cytometry-Based Assay to Detect Alpha Galactosidase Enzymatic Activity at the Cellular Level

**DOI:** 10.3390/cells13080706

**Published:** 2024-04-19

**Authors:** Nóra Fekete, Luca Kamilla Li, Gergely Tibor Kozma, György Fekete, Éva Pállinger, Árpád Ferenc Kovács

**Affiliations:** 1 Department of Genetics, Cell and Immunobiology, Semmelweis University, 1085 Budapest, Hungary; leona.fekete@gmail.com (N.F.); eva.pallinger@gmail.com (É.P.); 2 For Human Genome Foundation, 1094 Budapest, Hungary; 3 Pediatrics Centre, Tűzoltó Street Department, Semmelweis University, 1085 Budapest, Hungary; lilucakamilla17@gmail.com (L.K.L.); fekete.gyorgy@med.semmelweis-univ.hu (G.F.); 4 Nanomedicine Research and Education Center, Department of Translational Medicine, Semmelweis University, 1085 Budapest, Hungary; kozmalak@gmail.com; 5 SeroScience LCC, 1089 Budapest, Hungary

**Keywords:** Fabry disease, AGAL activity, flow cytometry, single cells, globotriaosyl–ceramide

## Abstract

Background: Fabry disease is a progressive, X chromosome-linked lysosomal storage disorder with multiple organ dysfunction. Due to the absence or reduced activity of alpha-galactosidase A (AGAL), glycosphingolipids, primarily globotriaosyl-ceramide (Gb3), concentrate in cells. In heterozygous women, symptomatology is heterogenous and currently routinely used fluorometry-based assays measuring mean activity mostly fail to uncover AGAL dysfunction. The aim was the development of a flow cytometry assay to measure AGAL activity in individual cells. Methods: Conventional and multispectral imaging flow cytometry was used to detect AGAL activity. Specificity was validated using the *GLA* knockout (KO) Jurkat cell line and AGAL inhibitor 1-deoxygalactonojirimycin. The *GLA* KO cell line was generated via CRISPR-Cas9-based transfection, validated with exome sequencing, gene expression and substrate accumulation. Results: Flow cytometric detection of specific AGAL activity is feasible with fluorescently labelled Gb3. In the case of Jurkat cells, a substrate concentration of 2.83 nmol/mL and 6 h of incubation are required. Quenching of the aspecific exofacial binding of Gb3 with 20% trypan blue solution is necessary for the specific detection of lysosomal substrate accumulation. Conclusion: A flow cytometry-based assay was developed for the quantitative detection of AGAL activity at the single-cell level, which may contribute to the diagnosis of Fabry patients.

## 1. Introduction

Lysosomal storage diseases (LSDs) are monogenic disorders in which the clinical symptoms are caused by pathogenic gene variants encoding for different lysosomal proteins, including lysosomal enzymes [1]. When a lysosomal enzyme is affected an excessive substrate accumulation due to the absence or impaired function of specific lysosomal hydrolase can be observed [2]. Fabry disease (MIM 301500) has an estimated incidence of 1:3000–7800, being the most prevalent LSD [3,4,5]. It is an X-linked inborn deficiency of alpha-galactosidase A enzyme (AGAL). From the genetic perspective, pathogenic variants in AGAL encoding the *GLA* gene are causative. Clinically manifested Fabry disease may occur if the AGAL activity decreases to 35% or less of the healthy state [6,7]. From a clinical perspective Fabry disease may be divided in two forms: the classical phenotype, with patients showing first symptoms typically in the first decade of life, and the more prevalent atypical form, with patients usually developing a more restricted symptomatology (e.g., mostly cardiovascular or renal manifestation or a subtle systemic manifestation) in the second or third decade of life. The symptomatology is associated with abnormal glycosphingolipid metabolism and the progressive lysosomal accumulation of the cytotoxic deacylated derivative of globotriaosyl-ceramide (globotriaosylsphingosine, lyso-Gb3) which has proinflammatory [8] and profibrotic [9] effects. Cardiac and other tissue-infiltrated lymphocytes and macrophages have been observed in Fabry patients, suggesting the contribution of these cells to the chronic inflammation observed in patients [10]. Fabry disease typically affects several organs, and has a progressive nature over time [11,12]. The clinical hallmarks of Fabry disease are (1) hypertrophic cardiomyopathy (signs of Gb3 accumulation detectable via decreased T1 signal, signs of myocardial fibrosis on heart-MRI, cardiac arrhythmias seen on ECG, abnormal wall motion on ECHO, elevated serum NT-proBNP), (2) renal dysfunction (reflected by podocyturia, microalbuminuria, proteinuria; glomerular and interstitial fibrosis, tubular atrophy resulting in decreased glomerular filtration rate), (3) dysfunction of the nervous system (acroparaesthesia due to small fiber neuropathy, neuropathic pain; basilar artery dolichoectasia, T1 hyperintensity—pulvinar thalami, T2 and FLAIR periventricular white matter lesion signs detectable on brain MRI), (4) skin-related symptoms (angiokeratomas, teleangiectasia, and hypo-/anhidrosis), (5) eye-related symptoms (cornea verticillata and retinal artery tortuosity). Based on a well-defined criterion system when three or more points out of six can be given, the clinical suspicion for Fabry disease is strong.

One point can be given for either (1) cornea verticillata and/or biopsy confirmed angiokeratoma; (2) if the plasma/leukocyte AGAL activity is below 5%; (3) if elevated Gb3 or lyso-Gb3 can be detected in plasma and/or urine; (4) if a likely pathogenic (American College of Medical Genetics and Genomics—ACMG class IV, or pathogenic—ACMG class V) *GLA* gene variant is detected. Two points may be given if a heart or kidney biopsy shows characteristic signs (e.g., lipid deposits in podocytes or lipid-laden lysosome) for Fabry disease [10,13,14,15,16]. Fabry disease belongs to the actionable genetic diagnosis group, as it is included in the secondary findings reporting of ACMG guidelines [17]. As a treatable genetic disorder, currently in several countries worldwide three approved intravenous biweekly administered enzyme replacement therapies are available: agalsidase alfa (Replagal, Takeda Pharmaceuticals International, Bannockburn, IL, USA), agalsidase beta (Fabrazyme, Sanofi Genzyme, Cambridge, MA, USA), and the recently approved pegunigalsidase alfa (Elfabrio, Chiesi Farmaceutici S. p. A, Parma, Italy). In selected Fabry patients with amenable pathogenic variants, the bidaily administered oral pharmacological chaperone therapy may be an alternative. Also, a gene therapeutical approach is under development, currently being in clinical trial Phase I/II (NCT02800070). Therefore, the early diagnosis and assessment for disease specific therapy eligibility to significantly halt the progression rate of Fabry disease is of critical importance [10,18].

In affected women, the fluorometry assay-based AGAL activity may be false negative in up to 40% of Fabry-affected women [19] due to the random X chromosome inactivation occurring during early embryogenesis. Therefore, in women the mean AGAL activity moves in a wide range from normal to very low, resulting in a limited screening value [10]. Affected Fabry females are mosaic to *GLA* expression, the severity of organ involvement in females being determined by the X chromosome inactivation pattern [20]. Skewed X chromosome inactivation occurring in female heterozygotes with a preference for pathogenic variant carrying *GLA* allele is associated with a higher phenotypic burden [10]. Moreover, the *GLA* sequencing in women may yield a variant of unknown significance (ACMG class III), leaving in a grey zone the diagnosis of Fabry disease [5].

Although AGAL activity can be measured in plasma, dried blood spots, and in isolated white blood cells, the leukocyte enzyme activity measurement is the gold standard [21,22]. The limitations of the assay are that the sample preparation method is time consuming and laboratory intensive, and the assay can only measure an average enzymatic activity. Therefore, the aim of our study was to develop a simple, rapid, flow cytometry-based AGAL enzyme activity measurement method that can detect the enzymatic activity at single-cell level.

## 2. Materials and Methods

### 2.1. Culturing of Jurkat Cell Line

Jurkat human acute T cell leukaemia cells (E6.1 clone, Merck KGAA, Darmstadt, Germany, RRID: CVCL_0367) were cultured in RPMI-1640 medium supplemented with 2 mM L-glutamine, 10% FBS, 100 U/mL penicillin, and 100 µg/mL streptomycin, in T25 flasks at 37 °C in humidified atmosphere, in the presence of 5% CO_2_. All chemical reagents were purchased from Merck Company (Merck KGAA, Darmstadt, Germany). Cell viability was assessed with CORNING automatic cell counter (Merck KGAA, Germany) using 0.4% trypan blue solution (Thermo Fisher, Waltham, MA, USA) in a 1:1 ratio and analyzed in CytoSmart software (version Cell Count-V3, CytoSMART Technologies, Eindhoven, The Netherlands). Jurkat cells were characterized by flow cytometry using anti-human CD3-PE (clone UCHT1, Sony Biotechnology Inc., San Jose, CA, USA) and anti-human CD4-APC (clone SK3, Sony Biotechnology Inc., San Jose, CA, USA) conjugated antibodies (Supplementary Figure S1A).

### 2.2. Mycoplasma Screening

Before transfection, Jurkat cells were screened by PCR-based mycoplasma screening test (PCR Mycoplasma Test Kit I/C, PromoCell, Heidelberg, Germany) according to the manufacturer’s instructions. Briefly, the Jurkat cells were centrifuged at 500× *g* for 5 min, and 1 mL of the supernatant was centrifuged at 14,000× *g* for 15 min, and the pellet was resuspended in 100 µL Dnase/Rnase free H_2_O. After 10 min of incubation at 95 °C, a 2 µL sample and 23 µL of rehydration buffer were added to the PCR tubes. A 40-cycle PCR program was used following a 2 min 95 °C activation, including 94 °C, 30 s followed by 55 °C, 30 s, and 72 °C, 40 s steps. The obtained PCR product was run on 1.5% agarose gel along with a DNA ladder and positive and negative controls (Supplementary Figure S1B).

### 2.3. Generation of GLA KO Jurkat Cells by CRISPR-Cas9 Genome Editing

The seven exons containing human *GLA* gene were localized at the Xq22 position. They encode the 429 amino acid long AGAL enzyme. To knockout the enzyme activity, 3 synthetic guide RNAs (sgRNA) were designed to target the 1st exon of the *GLA* gene (NC_000023.11 sgRNA1 UCACCGUGACAAUGCAGCU targeting g. chrX:101,407,883, sgRNA2 CAUCCCUGGGGCUAGAGCAC targeting chrX:101,407,797, sgRNA3 CUCCCAGUGCAGCCAGCCCA targeting g. chrX:101,407,794) using Synthego CRISPR Design Tool (Synthego Corporation, Redwood City, CA, USA). According to manufacturer’s instructions, sgRNAs were rehydrated in a 1xTE buffer to a concentration of 100 pmol/µL. A total of 1.5 × 10^5^ Jurkat cells, resuspended in 19.6 µL 82% SE solution containing 20 pmol Cas9 nuclease 2NLS, *S. pyogenes* (Synthego Corporation, Redwood City, CA, USA)/well along with the 3 sgRNAs using the CL-120 program were transfected using Nucleofector 4D-X (Lonza, Cologne, Germany). Mock transfection (without gRNA) was used to follow-up on potential effects of transfection reagents on the cells. After nucleofection, the cells were incubated at room temperature (RT) for 10 min, then 30 µL 20% FBS containing RPMI-1640 media was added onto the cells. Ten minutes afterwards, the cells were transferred to a 24-well cell culture plate (Eppendorf, Germany). Viability was assessed by flow cytometry (propidium iodide viability staining) and an automated cell counter (CORNING automatic cell counter) (Supplementary Figure S1C).

### 2.4. Clone Selection and Validation of GLA KO Jurkat Cells

Twenty-four hours after transfection, the total RNA was isolated with RNeasy Mini Kit (Qiagen, San Diego, CA, USA). The RNA concentration was determined with a Qubit 4 Fluorometer using the Qubit™ RNA HS Assay Kit (Thermo Fisher, Waltham, MA, USA). The Sensifast cDNA Synthesis Kit (Bioline, London, UK) was used for cDNA synthesis (500 ng RNA/sample). *GLA* expression was quantified with SYBR Green primers (GLA forward: GACTGGCAGAAGCATTGTGTACTC; *GLA* reverse: AAAATTTCGCCAGTGATTGC) and the SensiFAST SYBR Hi-ROX kit using the CFX Opus 384 real-time PCR instrument (Biorad, Hercules, CA, USA). The real-time PCR results were calculated according to the following formula: relative expression level = 2^−∆Ct,^ where ∆Ct = Ct (of gene of interest)—Ct (of housekeeping gene) with *HPRT* as an endogenous control.

Single-cell limited clone dilution was performed reaching a 1000 cells/mL as a working concentration from the transfected Jurkat *GLA* KO cells. One µL (containing a single cell) was pipetted into individual wells of a 96 well plate (Eppendorf, Germany) containing 50 µL of RPMI-1640 culture media with the supplementation as mentioned in the culturing of Jurkat cells. The wells containing single cells were identified with light microscopy, and only the cells seeded in them were used in the subsequent experiments. When the individual clones (Jurkat *GLA* KO and mock-transfected Jurkat cells) reached to approximately 1 × 10^5^ cells/clone, cells from the single clones were frozen and stored in 90% FBS and 10% dimethyl sulfoxide (DMSO) at −80 °C in 3 aliquots/clone. Afterwards, 10 clones were thawed, following RNA extraction and reverse transcription *GLA* expression was determined as described before. In one clone, where *GLA* could not be detected, a further aliquot was thawed and DNA was extracted using the QIAamp DNA Mini Kit (Qiagen, Hilden, Germany). Library preparation was performed using IDT xGen2.0 with UDIs and the 2 × 150 bp paired sequencing was done on a NovaSeq 6000 instrument. The mean region coverage was 120 and the Q30 value was 94%. The data were aligned to hg38, and the on-target *GLA* gene sequence was evaluated in the Jurkat *GLA* KO and Jurkat mock-transfected cell lines. Afterwards, in silico off-target analysis was performed. All genomic regions where possible off-targets could be found according to the in silico analysis were evaluated by a whole exome sequencing approach, and analyzed using Genome Browser (Golden Helix, Bozeman, MT, USA).

### 2.5. Flow Cytometric Monitoring of Alpha-Galactosidase A Enzyme Activity

The AGAL activity of the Jurkat cells was assessed by using the N-Dodecanoyl-NBD-ceramide trihexoside (Abcam, Cambridge, UK) fluorescent glycosphingolipid analog (Fl-Gb3). Fl-Gb3 is comparable to natural trihexoside in many biological functions. It is also the substrate of alpha-galactosidase; therefore, it is usable for the detection of AGAL activity [23]. Fl-Gb3 stock substrate was dissolved in DMSO for preparing a 300 nmol/mL stock solution according to the manufacturer’s instruction.

Enzyme activity measurements were started with a kinetic detection in order to determine the required incubation time for the endpoint measurements. Jurkat cells in log-phase growth were pelleted and resuspended at 1 × 10^6^ cells/mL in RPMI-1640 medium (Thermo Fisher Scientific, USA) supplemented with 10% FBS (Merck KGAA, Germany). To assess the AGAL activity, 1 × 10^5^ Jurkat cells were seeded into 96-well plates and incubated for different times (5 min, 1 h, 3 h, 5 h, 6 h, 8 h, 16 h and 24 h) with a substrate working solution (final concentration in 2.83 nmol/mL) at 37 °C in the dark, in 96-well plates. Before the FACS measurements, the cells were washed twice and were resuspended in trypan blue solution (end concentration 0.2%) in order to quench the exofacial attached fluorescence substrate [24,25]. The specificity of the enzyme activity measurement was confirmed by additional incubation of samples with a specific AGAL inhibitor, the 1-deoxygalactonojirimycin (DGJ) [26] at 500 μM of final concentration in parallel with the addition of FL-Gb3. FACS measurements were carried out by CytoFLEX S Flow Cytometer (Beckman Coulter Life Sciences, Brea, CA, USA) equipped with a blue laser with an excitation of 488 nm. Substrate fluorescence was monitored in the FL1 channel at 525/40 nm. CytExpert Software 2.4 (Beckman Coulter Life Sciences, Brea, CA, USA) and Flowjo 9.6.1 software were used for analysis. The gating strategy based on the definition of Jurkat cells: (1) on FSC/SSC dot plot (“morphology”), (2) the single cells (FSC-A/FSC-H dot plot), (3) viable cells by trypan blue staining (Supplementary Figure S2A).

### 2.6. Imaging Cytometry for the Validation of AGAL Enzymatic Activity

Imaging flow cytometry was performed on an ImageStreamX Mark II operated by INSPIRE software (version 201.1.0.765, Amnis Corporation, Seattle, WA, USA). For bright-field (channel: 01) and side-scatter (channel: 06) image capturing, blue-colored LED lighting and a 785 nm laser were used, respectively. N-Dodecanoyl-NBD-ceramide trihexoside substrate and trypan blue fluorescence were recorded using excitation with a 488 nm laser at 200 mW intensity and the emissions were collected with a 533/55 nm–filter (“green substrate” channel: 02) and with a 702/85 nm–filter (“Trypan blue” channel: 05). A total of 5000 events were acquired for each sample. Unstained controls and single-color control samples (without bright field and SSC excitation) were used to determine autofluorescence and to calculate a spectral crosstalk matrix.

### 2.7. Statistical Analysis

Graphpad Prism 9.0 (Graphad, San Diego, CA, USA) was used for statistical analysis. Data showing a normal distribution an unpaired Student’s *t*-test, for a multiparametric analysis ANOVA test following Bonferroni correction, were used. Data showing a not normal distribution a Mann–Whitney U test or Wilcoxon test were used. Statistical significance was set to *p* < 0.05.

## 3. Results

We divided the development of the single-cell resolution AGAL flow cytometric assay into two steps: (1) we optimized the flow cytometric detection of Fl-Gb3 in Jurkat lymphoblastic cells and (2) by using a reversible AGAL inhibitor and creating a *GLA* KO cell line, we proved that the enzyme activity detected with Fl-Gb3 substrate refers to the AGAL enzyme.

### 3.1. Flow Cytometric Detection of Alpha-Galactosidase A Enzyme Activity

**Figure 1:**
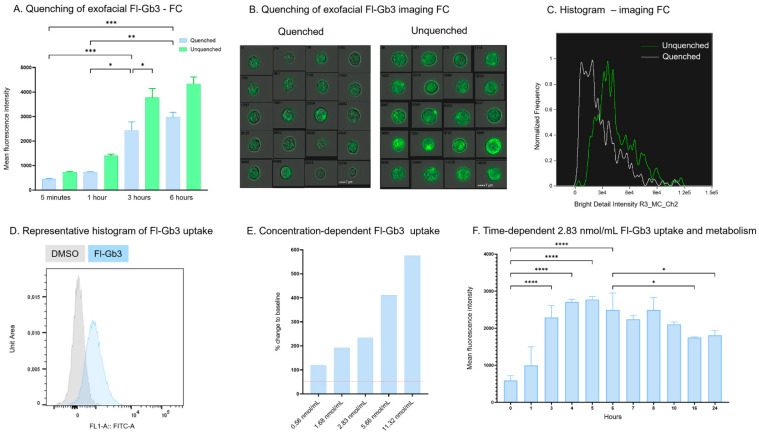
Detection of AGAL activity with flow cytometry. (**A**) Quenching of the 2.83 nmol/mL exofacial Fl-Gb3 allows the detection of intracellular Fl-Gb3 detection via flow cytometry. (**B**) Representative images of brightfield and masked cells (exofacial signal quenched via 0.2% trypan blue) showing the effect of quenching using imaging cytometry upon addition of 2.83 nmol/mL Fl-Gb3. (**C**) The validation quenching of exofacial Fl-Gb3, as the bright detail intensity decreases upon trypan blue quenching, as detected by imaging cytometry—green line unquenched cells, gray line quenched cells (**D**) Representative histogram of 2.83 nmol/mL exogenous Fl-Gb3 substrate accumulation—this resulted from measuring the fluorescent intensity of uptaken and hydrolysed Fl-Gb3 compared to cells treated with DMSO. (**E**) Concentration-dependent Fl-Gb3 metabolism change compared to baseline after 3 h of incubation with 0.56–11.32 nmol/mL Fl-Gb3, as shown. (**F**) Time-dependent curve showing a peak of 2.83 nmol/mL Fl-Gb3 uptake at 5 h incubation. Data are represented as mean ± SD; * *p* < 0.05, ** *p* < 0.01, *** *p* < 0.001, **** *p* < 0.0001 by one-way ANOVA followed by Tukey’s multiple comparisons test (**A**,**F**) or Dunnett’s multiple comparisons test (**E**). AGAL—alpha galactosidase A, FC—flow cytometry, Fl-Gb3—fluorescently labelled globotriaosyl-ceramide, DMSO—dimethyl sulphoxide.

The membrane-permeable Fl-Gb3 substrate analog was used for the determination of AGAL activity at the single-cell level after quenching the fluorescent signal derived from the surface-bound Fl-Gb3 by trypan blue solution (Figure 1A). Trypan blue quenching allows the differentiation of intracellular Fl-Gb3 substrate accumulation from the exofacial Fl-Gb3 signal (quenched 2434 ± 914.5 MFI vs. unquenched 3776 ± 1172 MFI, *p* = 0.012).

The cellular uptake detection of 2.83 nmol/mL Fl-Gb3 was also validated by imaging flow cytometry (Figure 1B,C). The detected fluorescence is determined by the amount of Fl-Gb3 accumulation and AGAL activity (Figure 1D). Therefore, the reduction in fluorescent signal is proportional with alpha-galactosidase enzymatic activity. Since no data are yet available on the use of the Fl-Gb3 substrate for flow cytometric measurement, the required incubation time and substrate concentration were titrated. Titration of the substrate concentration (from 0.56 nmol/mL to 11.32 nmol/mL range) resulted in an optimal measurement at the 2.83 nmol/mL final substrate concentration resulting in a 342 ± 108.1% increase compared to baseline fluorescent intensity (Figure 1E). In order to standardize the endpoint AGAL activity measurement, kinetic measurements were performed on a wide time interval (0 h–24 h). The peak fluorescent intensity could be detected after 5 h of incubation (2776 ± 80.79 MFI). A decrease in Fl-Gb3 could be observable after 6 h of incubation (2495 ± 494 MFI), a significant trend that could be detected in the 24 h observation period. According to our results, the balance of fluorescent substrate accumulation and enzymatic degradation shifted in the direction of substrate uptake in the first 5 h. After 6 h of incubation, substrate reduction dominates as a result of enzymatic degradation. We concluded that the 6 h incubation time is optimal for the end-point measurement of AGAL activity (Figure 1F).

### 3.2. Generation of the GLA KO Cell Line

**Figure 2:**
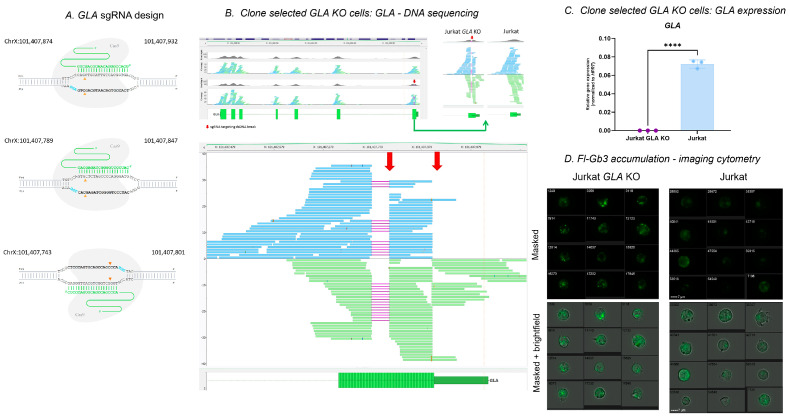
Generation of alpha galactosidase deficient Jurkat cells. (**A**) Guide RNA (sgRNA) design targeting the 1st exon of the *GLA* gene. (**B**) *GLA* gene editing analyzed at the DNA level with next-generation whole exome sequencing of transfected and wild-type Jurkat cells. (**C**) Analysis of *GLA* gene expression in transfected and wild-type Jurkat cells. (**D**) Detection of 2.83 nmol/mL Fl-Gb3 substrate accumulation after 3 h incubation, as detected by imaging cytometry showing masked and corresponding brightfield included *GLA* KO and wild-type Jurkat cells. Data are represented as mean ± SD; **** *p* < 0.0001 by Student’s *t* test (**C**). Fl-Gb3—fluorescently labelled globotriaosyl-ceramide; KO—knockout.

In order to prove the specificity of Fl-Gb3-associated AGAL enzyme activity measurements, we created a *GLA* KO Jurkat cell line using the CRISPR/Cas9 genome editing system. We designed guide RNAs (sgRNA) targeting the 1st exon of the AGAL encoding *GLA* gene (Figure 2A). This was followed by nucleofection-based transfection and single-cell cloning and expansion. The generated *GLA* KO cell line carries a 36 base pair deletion in the 1st exon of the GLA gene: seq[GRCh38] NC_000023.11(NM_000169):g.101,407,776_ 101,407,812del (Figure 2B). The transfection efficiency was validated also at RNA level, with undetectable *GLA* expression in *GLA* KO cell line (Figure 2C). The absent enzyme activity was visualized by imaging flow cytometry (Figure 2D).

### 3.3. Validation of the Specificity of the AGAL Enzyme Activity Measurement with a Reversible AGAL Inhibitor (1-Deoxygalactonojirimycin)

**Figure 3:**
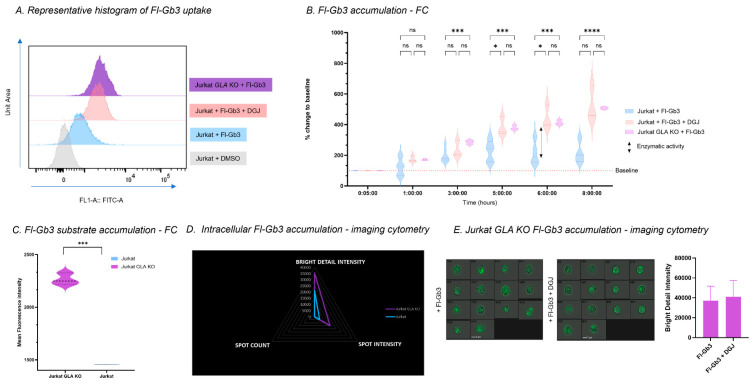
Validation of specific AGAL activity detection. (**A**) Fl-Gb3 substrate accumulation can be detected upon 2.83 nmol/mL Fl-Gb3 addition to Jurkat cells (blue histogram), treatment of wild-type Jurkat cells with 2.83 nmol/mL Fl-Gb3 and DGJ (rose histogram) yields the same accumulation as GLA KO Jurkat cells treated with 2.83 nmol/mL Fl-Gb3 (purple histrogram). (**B**) Upon treatment with 2.83 nmol/mL Fl-Gb3 a time-dependent substrate accumulation can be detected in AGAL deficient Jurkat cells, similarly to wild-type Jurkat cells treated with AGAL inhibitor (bullets show mean of 50,000 cells/measurement). (**C**) Fl-Gb3 substrate accumulation is significantly higher in *GLA* KO Jurkat cells. (**D**) Radar plot showing the quantification of significantly higher Fl-Gb3 accumulation in GLA KO Jurkat cells of spot intensity count, spot count, and bright detail intensity, respectively. (**E**) Fl-Gb3 treated Jurkat GLA KO cells show no difference upon addition of DGJ, as shown in masked brightfield images, as well in bar plot, summarizing the bright detail intensity detected by imaging cytometry. Data are represented as mean ± SD; * *p* < 0.05, *** *p* < 0.001, **** *p* < 0.0001 by two-way ANOVA followed by Tukey’s multiple comparisons test (**B**) or Student’s *t* test (**C**). FC—flow cytometry; AGAL—alpha galactosidase A; Fl-Gb3—fluorescently labelled globotriaosyl-ceramide; DGJ—1-deoxygalactonojirimycin; KO—knockout; ns—not significant.

Five hundred µM of DGJ-competitive AGAL inhibitor was used to prove the specificity of enzyme activity measurements, both in the case of flow cytometric and imaging cytometric measurements. In the presence of DGJ inhibitor, a significantly higher fluorescence signal could be detected due to the reduced enzyme activity after 3, 5, 6, or 8 h of incubation (Figure 3B).

### 3.4. Detection of Specific AGAL Activity in GLA KO Cells

Using the results of the AGAL activity measurement set on wild-type Jurkat cells, we examined the enzyme activity of *GLA* KO cells. Incubation of *GLA* KO Jurkat cells with 2.83 nmol/mL Fl-Gb3 showed the same pattern of substrate accumulation as the DGJ-specific AGAL inhibitor-treated WT Jurkat cells. After 3 h of incubation, a consistent and significant increase in *GLA* KO Jurkat cells could be observed compared to wild-type Jurkat cells (wild type 194.5 ± 41.51% vs. DGJ-treated 235.4 ± 53.29% vs. *GLA* KO 285.4 ± 13.12% to baseline, *p* < 0.001), this was most prominent after 6 h of incubation (wild type 216.4 ± 73.82% vs. DGJ-treated 442.8 ± 75.51% vs. *GLA* KO 413.3 ± 16.18% to baseline, *p* < 0.001) (Figure 3B). Of note, no significant difference between DGJ-treated wild-type Jurkat cells and *GLA* KO Jurkat cells could be detected in Gb3 accumulation regardless of the incubation period. Comparing the wild-type Jurkat cells to *GLA* KO Jurkat cells, a significant increase in Fl-Gb3 could be detected via flow cytometry (*GLA* KO 2256 ± 56.03 MFI vs. wild type 1457 ± 1.41 MFI, *p* < 0.001) (Figure 3C) and imaging cytometry (Figure 3D). Furthermore, no difference in fluorescence intensity could be detected upon DGJ addition of Fl-Gb3-treated Jurkat GLA KO cells (Figure 3E).

## 4. Discussion

The measurement of AGAL activity is a critical point in the diagnosis of Fabry disease, which has therapeutic consequences. In the case of female patients, the heterogeneous and not always clear initial symptoms may delay the establishment of the diagnosis. The currently available methods are labor-intensive (the working processes consisting of several steps), which cannot be fulfilled in all clinical laboratories. The aim of this work was to develop a fast, flow cytometry-based, less labor-intensive AGAL activity method which is widely applicable.

Deficient AGAL activity results in elevated levels of glycosphingolipids, therefore the uptake and the breakdown of synthetic N-Dodecanoyl-NBD-ceramide trihexoside fluorescent glycosphingolipid analog (Fl-Gb3) can be characteristic to AGAL enzyme activity. As Fl-Gb3 can also bind to cell surfaces, we applied the quenching of the exofacial fluorescent signal. AGAL enzyme activity was validated by two different methods: (1) by the using a specific AGAL inhibitor, the 1-deoxygalactonojirimycin (DGJ), and (2) by the using *GLA* knockout (KO) Jurkat cells, created by the CRISPR/Cas9 gene editing method. Flow cytometric analysis was visualized by imaging flow cytometry.

The quenching of exofacial Fl-Gb3 was obtained with addition of 0.4% trypan blue before sample measurement. Trypan blue acts as a non-penetrating quencher and can successfully quench the green fluorescence attached to the cell membrane and enables the detection of internalized compound [27]. To detect endogenous AGAL activity, along the exogenously added fluorescent substrate the reversible inhibitor was also added. DGJ acts via binding to AGAL (IC_50_ = 79 nM) using non-covalent interactions. The endocyclic nitrogen of DGJ becoming protonated forms an ion pair with the negatively charged amino acid residue of the AGAL active site [10]. Therefore, in cells incubated with Fl-Gb3, the active AGAL binds the exogenously added substrate and after 6 h of incubation, a significant degradation of the substrate is elicited. In contrast, the DGJ-blocked cells will not degrade the substrate, thus via comparison of Fl-Gb3 to Fl-Gb3 + DGJ, the fluorescent ratio permits a precise, single-cell resolution measurement of AGAL activity.

AGAL activity may be influenced by several cellular environmental and metabolism factors, e.g., lysosomal stability, pH stability, and temperature stability. Also, AGAL activity shows cell-type specific variations [28]. The difference in cellular states during cell culturing may explain the higher variation observable in our in vitro experiments in wild-type Jurkat cells. This hypothesis is further confirmed by the low variation observable in the *GLA* KO Jurkat cell line, as there is a complete absence of AGAL activity in this scenario.

Although several newborn sequencing-based programs and trials have started worldwide [29,30,31] to assess the benefit or early diagnosis, regarding the evaluation of variants of the *GLA* gene from whole genome sequencing data in the analysis pipeline is not included in a significant part of the trials, mostly due to the uncertain clinical action upon detection of a variant of unknown significance of certain *GLA* gene variants, as well the uncertainty of the clinical impact of certain (likely) pathogenic variants. As for future perspectives, the detection of *GLA* transcript variants, deep intronic variants and an aberrant methylation pattern via third-generation nanopore sequencing could also assist the molecular diagnosis, where a deficient enzymatic activity could be observed at single-cell resolution [32]. The detection of AGAL activity at a single-cell level and the assessment of intercellular variations in enzymatic activity could both boost understanding and pave the way for supporting the decision of clinically uncertain *GLA* gene variants in asymptomatic patients or in affected individuals showing only a subtle, mostly nonspecific phenotypic burden.

## 5. Conclusions

We have developed a fast and easy flow cytometry-based assay to quantify alpha galactosidase activity at the single-cell level by using a fluorescent AGAL substrate analog. We proved the specificity of our assay at multiple levels, including by a reversible enzyme inhibitor and development of a *GLA* KO model cell line. Our method is suitable for rapid determination of AGAL activity, so it can be adapted as a screening test for Fabry disease.

## Data Availability

Sequencing data of the Jurkat *GLA* KO cell line can accessed via project accession PRJEB71791, deposited in the European Nucleotide Archive (ENA).

## Supplementary Materials

The following supporting information can be downloaded at: https://www.mdpi.com/article/10.3390/cells13080706/s1, Figure S1: mycoplasma test and cell viability; Figure S2: gating strategy.
