# Supplementary material for: Flow Cytometry-Based Assay to Detect Alpha Galactosidase Enzymatic Activity at the Cellular Level
Source: Cells. 2024 Apr 19;13(8):706. doi: 10.3390/cells13080706 (PMC11049294; doi:10.3390/cells13080706)
Supplement: Supplementary file 1 [file cells-13-00706-s001.zip › cells-2897071-supplementary.pdf]

Suppl. Fig. 1.

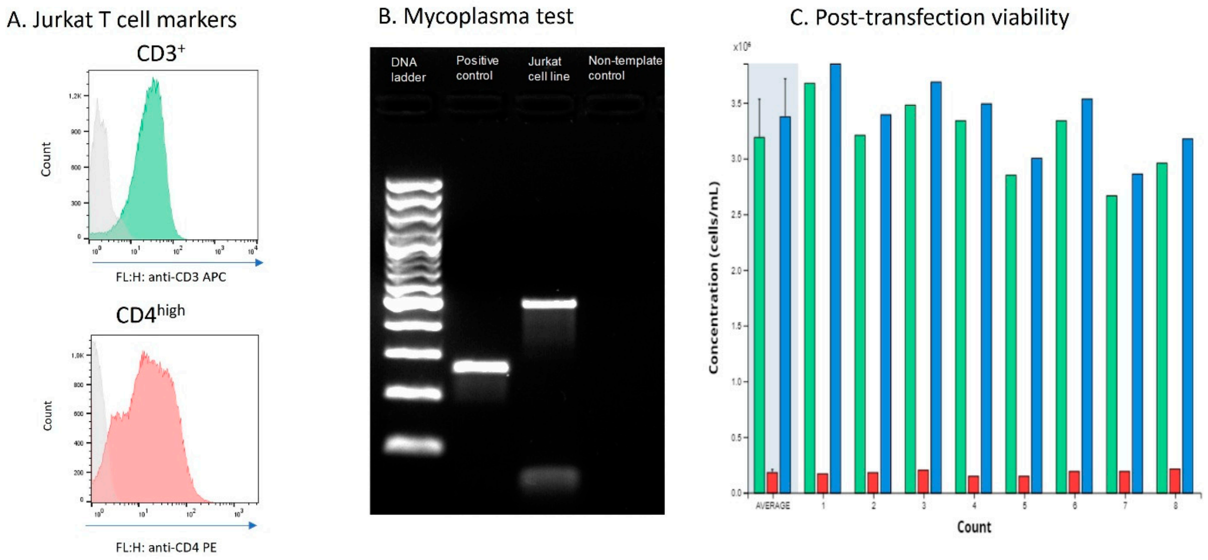

Suppl.Fig.1. Pre-transfection characterization of Jurkat cells and post-transfection viability of *GLA* KO Jurkat cells. **A**. Jurkat cells expressed CD3 and CD4 cell surface markers **B**. Jurkat cells were not contaminated by Mycoplasma spp. **C**. The viability of transfected *GLA* KO Jurkat cells 24 hours after transfection was above 95% (green bar- concentration of viable cells, red bar- concentration of dead cells, blue line – concentration of cell count). KO – knockout.

Suppl. Fig. 2.

Gating strategy

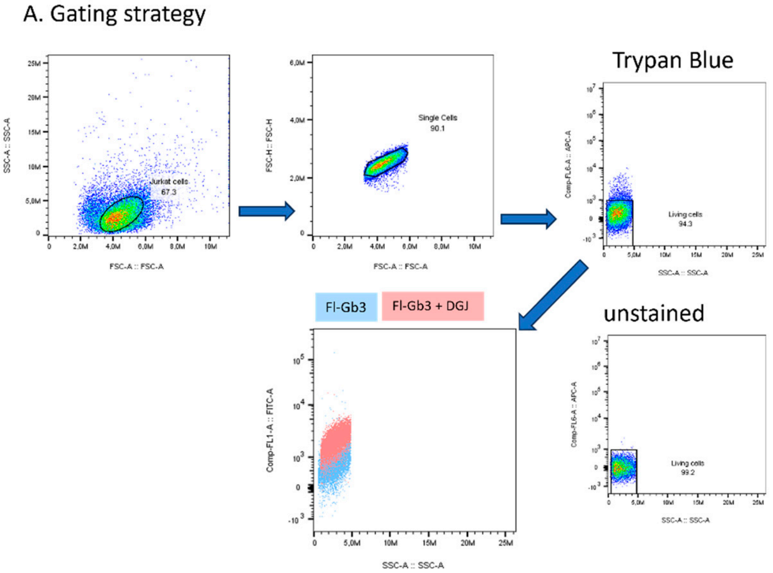

Suppl.Fig.2. Gating strategy and effect of trypan blue quenching for AGAL enzymatic activity measurement. A. Morphologically intact Jurkat cells were gated, followed by single cell gate, afterwards a third gate of living cells (trypan blue negative cells) was applied. Enzymatic activity detection is based on the fluorescent channel 1 (FITC) signal intensity difference between fluorescently labelled globotriaosyl-ceramide (Fl-Gb3) incubated cells and reversible AGAL inhibitor containing Fl-Gb3 incubated cells. AGAL – alpha galactosidase A, Fl-Gb3 – fluorescently labelled globotriaosyl-ceramide, DGJ - 1-deoxygalactonojirimycin.
